# *Salmonella* reduces tumor metastasis by downregulation C-X-C chemokine receptor type 4

**DOI:** 10.7150/ijms.60439

**Published:** 2021-06-01

**Authors:** Tai-Huang Lee, Gaun-You Lin, Ming-Hui Yang, Yu-Chang Tyan, Che-Hsin Lee

**Affiliations:** 1 Department of Internal Medicine, Kaohsiung Municipal Ta-Tung Hospital, Kaohsiung Medical University Hospital, Kaohsiung Medical University, Taiwan.; 2 Department of Biological Sciences, National Sun Yat-sen University, Kaohsiung, Taiwan.; 3 Department of Medical Education and Research, Kaohsiung Veterans General Hospital, Kaohsiung, Taiwan.; 4 Department of Medical Imaging and Radiological Sciences, Kaohsiung Medical University, Kaohsiung, Taiwan.; 5 Department of Medical Research, China Medical University Hospital, China Medical University, Taichung, Taiwan.; 6 Department of Medical Laboratory Science and Biotechnology, Kaohsiung Medical University, Kaohsiung, Taiwan.; 7 International Ph.D. Program for Science, National Sun Yat-sen University, Kaohsiung, Taiwan.

**Keywords:** *Salmonella*, Metastasis, Migration, CXCR4, CXCL12

## Abstract

Tumor metastasis is the main reason for the death of most cancer patients. C-X-C chemokine receptor type 4 (CXCR4) has been demonstrated to be overexpressed in numerous types of cancer. CXCR4 selectively binds with stromal cell-derived factor 1 (SDF1), also known as C-X-C family chemokine ligand 12 (CXCL12) (CXCL12/SDF-1), which induced tumor proliferation and metastasis. Recently, the use of conventional cancer treatments had some limitation; bacteria treatment for cancer becomes a trend that overcomes these limitations. Plenty of studies show that *Salmonella* has anti-tumor and anti-metastatic activity. The current study aimed to investigate *Salmonella* suppresses CXCR4 protein expression and tumor cell migration ability in B16F10 melanoma and LL2 lung carcinoma cells. *Salmonella* reduced CXCR4 protein expression through downregulating Protein Kinase-B (Akt)/Mammalian Target of Rapamycin (mTOR) signaling pathway. In cells transfected with constitutively active Akt plasmids, a reverse effect of *Salmonella*-induced inhibition of CXCR4 was observed. Tumor cells have chemotactic response to CXCL12 in migration assay, and we found that *Salmonella* reduced tumor chemotactic response after CXCL12 treatment. The C57BL/6 mice were intravenously injected with B16F10 and LL2 cells pre-incubated with or without *Salmonella*, the tumor size and lung weight of *Salmonella* group had obviously decreased, indicating anti-metastatic effect that confirmed the findings from the* in vitro* experiments.

## Introduction

The C-X-C chemokine receptor type 4 (CXCR4)/ stromal cell-derived factor 1 (SDF-1) axis has been reported to participate in the proliferation, migration, and invasion of several cancer types 1. Metastatic spread of most cancers, including melanoma, lung cancers, generally leads to high mortality rate despite available interventions 2. The CXCR4 in tumors correlates with tumor size, metastasis, and prognosis 3. CXCR4 phenotype knockout strategies have been widely used to inhibit tumor metastasis mediated by the CXCR4/SDF-1 axis 3. CXCR4 expression was reported to be mediated by several signaling mechanisms including protein kinase B (Akt) signaling that is known to modulate behavioral phenotypes in tumor such as anti-apoptosis, drug resistance and metastasis 4. Thus, activation of the SDF-1/CXCR4/AKT pathway is important for tumor metastases and progression 5.

*Salmonella*, the Gram-negative, facultative anaerobic bacteria, has being used in cancer therapy 6.* Salmonella* was used as tumor targeting therapeutic agents, because *Salmonella* was able to target small metastatic and primary tumors 7. Previously, we showed that* Salmonella* can colonize small tumor nodules and enhance the survival of lung metastatic tumor mice 8. In mouse metastatic tumor model, *Salmonella* can reduce the expression of Matrix metallopeptidase 9 (MMP-9) in tumors 9. Herein, we identified *Salmonella* as a key factor in decreasing the migration of tumor cells. We also explored a therapeutic strategy to decrease the expression of CXCR4.

## Materials and Methods

### Bacteria, Reagent, Cells, Plasmids, and Animals

A vaccine strain of Salmonella choleraesuis [S. choleraesuis subsp. choleraesuis (Smith) Weldin serovar Dublin (ATCC 15480)] was obtained from Bioresources Collection and Research Center (Hsinchu, Taiwan) 10. The resveratrol and 4',6-Diamidino-2-Phenylindole (DAPI) and mouse CXCL12/SDF-1 protein were purchased from Sigma-Aldrich (Sigma Aldrich, St. Louis, MO, USA). AMD3100 was purchased from Selleckchem (Sekkeckchem, Houston, TX, USA). Murine melanoma cells (B16F10) and murine lung cancer cells (LL2) were maintained in the Dulbecco's Modified Eagle's Medium (DMEM) containing 10% fetal bovine serum gentamicin (50 μg/mL). A constitutively active AKT plasmid was a kind gift from Dr. C-Y Tsai. (Department of Molecular Immunology, Osaka University) 11. The C57BL/6 mice were obtained from the National Laboratory Animal Center of Taiwan provided. The Laboratory Animal Care and Use Committee of the National Sun Yat-sen-University approved the experimental protocol (permit number: 10829).

### Western Blotting and Transfection

The protein content was used a bicinchoninic acid (BCA) protein assay (Pierce Biotechnology, Rockford, IL, USA) to determine the protein concentration. The sodium dodecyl sulfate polyacrylamide gel electrophoresis (SDS-PAGE) was used to fractionate protein samples. The protein samples were transfer to the hybond-enhanced chemiluminescence nitrocellulose membranes (Pall Life Science, Glen Cove, NY, USA). The membranes were incubated with various antibodies, including CXCR4 (GeneTex Inc. Irvine, CA, USA), the protein kinase B (AKT) (Santa Cruz Biotechnology, Santa Cruz, CA, USA), phosphorylation-AKT (Santa Cruz Biotechnology), mammalian targets of rapamycin (mTOR) (Cell Signaling, Danvers, MA, USA), phosphorylation-mTOR (Cell Signaling), p70 ribosomal S6 kinase (p-p70S6K) (Cell Signaling), phosphorylation-p70S6K (Cell Signaling), and β-actin (Sigma-Aldrich). The appropriate horseradish-peroxidase-conjugated secondary antibodies were used and enhanced chemiluminescence system (T-Pro Biotechnology, New Taipei City, Taiwan) to detect the protein-antibody complexes. Lipofectamine 2000 (ThermoFisher Scientific, Waltham, MA, USA) was used to transfect with the constitutively active AKT plasmids to cells.

### Wound-healing and Transwell Assays

The wound-healing according to the manufacturer's instructions (IBIDI, Martinsried, Germany). The moving distance was detected after 24 h by using a microscope. The migration distances of untreated cells were set to 100% and were compared with cells treated with* Salmonella*. The cell migration according to the manufacturer's instructions (Transwell cultures (ThermoFisher Scientific). Cells were stained with DAPI and counted under a fluorescence microscope 9.

### Mouse Experiments

The B16F10 (10^5^) and LL2 cells (10^5^) mixed with or without* Salmonella* (Multiplicity of infection, MOI = 100) for 1.5 h and C57BL/6 mice were injected with *Salmonella*-treated or non-treated-cells via the tail vein on Day 0. Tumor-bearing mice were sacrificed, and the lungs were removed, and weighed on day 18.

### Statistical analysis

Statistical analyses were performed by one-way ANOVA and Sidak's multiple comparison test relative to control groups using Prism 9. Any P value less than 0.05 is regarded statistically significant.

## Results

### *Salmonella* reduced the migration of tumor cell

**Figure 1:**
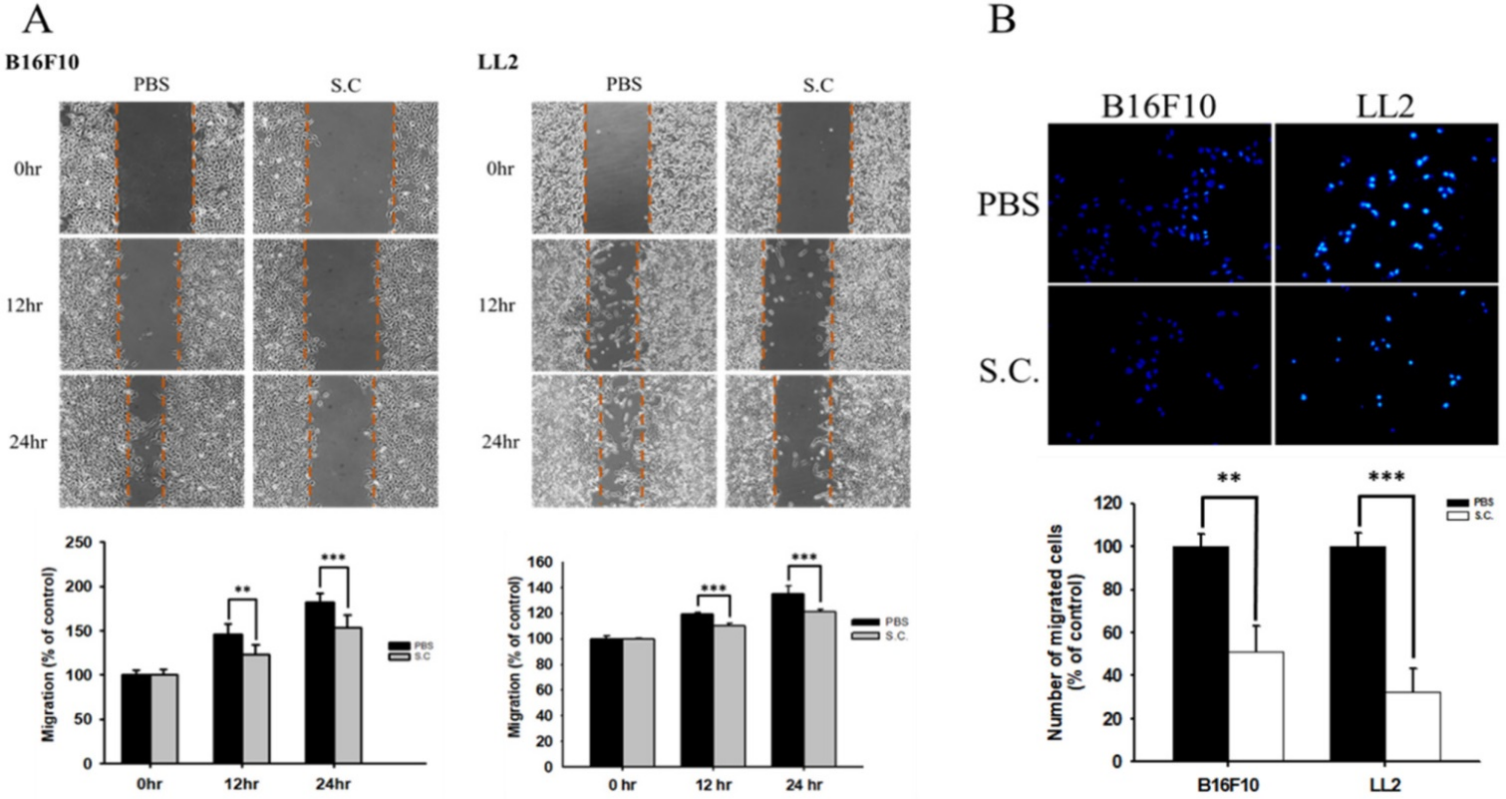
The cellular motility of B16F10 and LL2 cells after *Salmonella* (S.C.) treatment. The cells were co-cultured with *Salmonella* (MOI = 100) for 1.5 h. The motility distances of different groups of (**A**) B16F10 and LL2 cells were measured at different time points. (**B**) The B16F10 cells and LL2 cells were placed on the upper layer of Tranwell and then infected with *Salmonella* (MOI = 100) for 90 min. After 24 h, the bottom layer of cells were stained with 4',6-diamidino-2-phenylindole (DAPI) and counted under a fluorescence microscope (n = 6, mean ± SD. ** p < 0.01; *** p < 0.001). These replicates were used different passage of cells.

**Figure 2:**
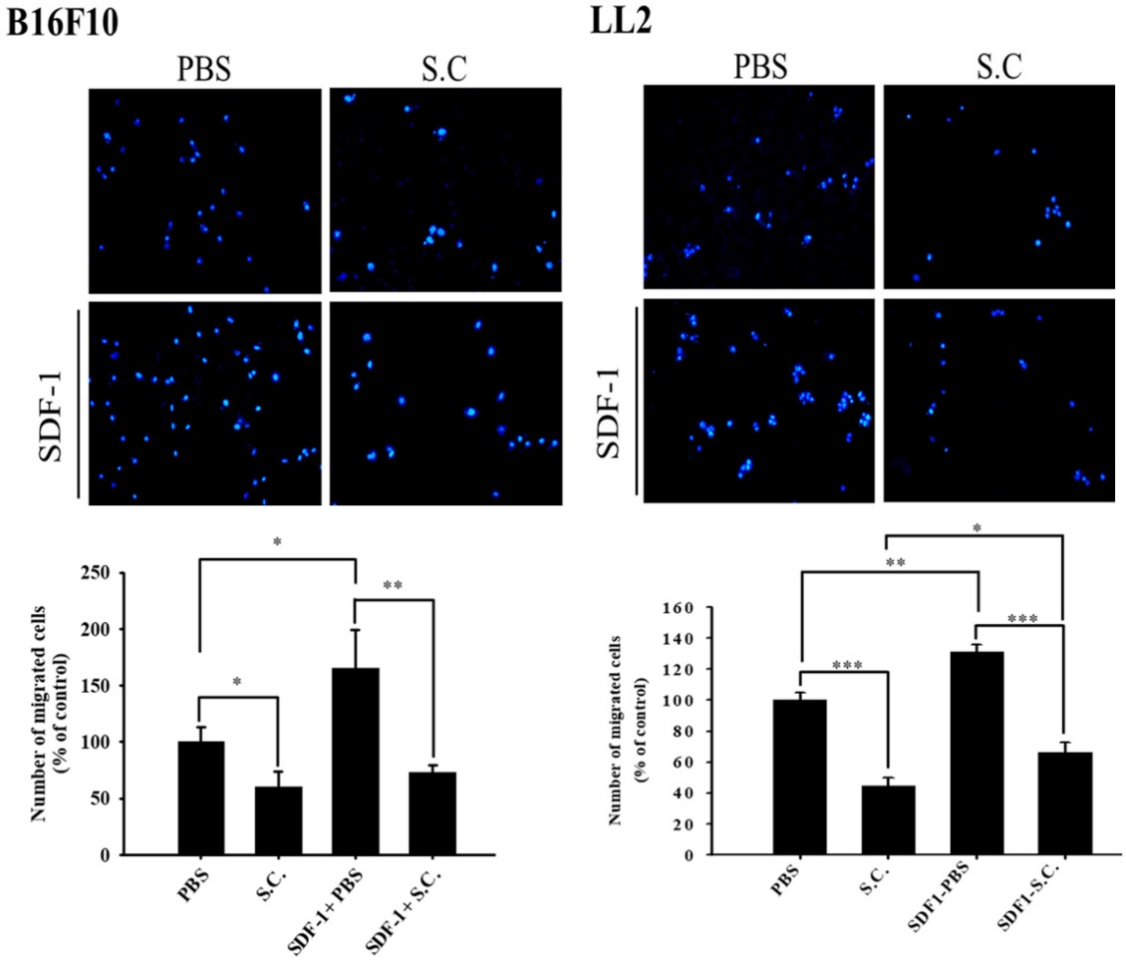
Tumor cells infected with *Salmonella* (MOI=100) were added to the upper chamber (with permeable membrane) in the 24-well plates and CXCL12 (100 ng/ml) were added in the lower chamber, then cultured for 24 hours. The cells which migrated through the permeable membrane were stained with DAPI and measured by fluorescensce microscope (200X) (n = 6, mean ± SD. *p < 0.05; ** p < 0.01; *** p < 0.001). These replicates were used different passage of cells.

The B16F10 mouse melanoma cells and LL2 mouse lung tumor cells were treated with* Salmonella* to examine the migration of tumor cells. The results of the wound-healing assay showed that the movement of tumor cells (B16F10 and LL2) was significantly inhibited compared with that of the PBS group after *Salmonella* treatment (Fig. 1A). The reduction of the motility of* Salmonella*-treated tumor cells was observed in Fig. 1A. Although *Salmonella* did not affect cell proliferation after a short period of infection, we further used Transwell assay to measure the migration of B16F10 and LL2 cells (Fig. 1B). As Fig 1B shown,* Salmonella* significantly reduced the movement of both types of tumor cells. Multiple tumor cell rely on CXCR4 and its ligand, SDF-1/CXCL12, to metastasis 12. Meanwhile, CXCR4 inhibitor (AMD3100) suppressed tumor cell migration as *Salmonella* (Fig. S1). It is well known that high concentration of SDF1 suppressed chemotaxis. As Fig. S2 and Fig. 2 shown, *Salmonella* inhibited SDF-1-induced tumor cell migration by Transwell assay. Our data showed that *Salmonella* reduces the motility of tumor cells.

### *Salmonella* dose-dependently inhibited CXCR4 expression in tumor cells

**Figure 3:**
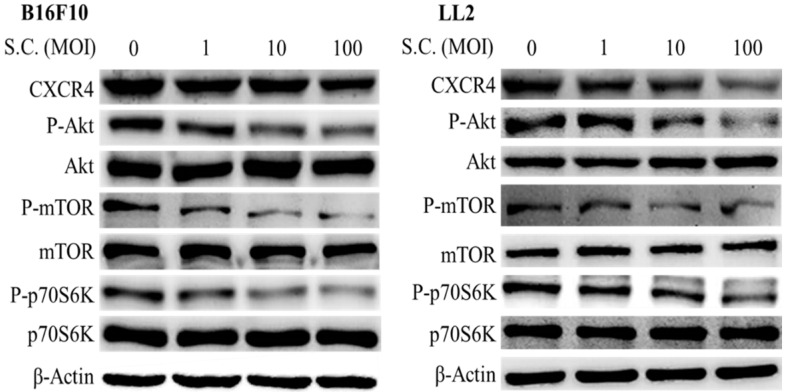
The CXCR4 expression in Salmonella-treated-B16F10 and-LL2 cells. The cells were co-cultured with *Salmonella* (MOI = 0-100) for 1.5 h. The protein expression in B16F10 and LL2 cells was measured. The immunoblotting assay was repeated three times with similar results. Inserted values indicated relative proteins expression in comparison with β-actin.

In this study, we found that* Salmonella* can inhibit SDF-1-induced tumor cell migration (Fig. 2). Furthermore, whether *Salmonella* can reduce tumor cell metastasis by inhibiting the receptor of SDF-1 (CXCR4), the protein expression in* Salmonella*-treated tumor cells were measured. We used Western blotting to evaluate the potential signaling pathways through which *Salmonella* has its anti-migration effects and examine the expression of CXCR4 and related signaling pathways (Fig. 3). The AKT/mTOR signaling is required for SDF-1 mediated migration in gastric carcinomas 13. It is surprising that we found that *Salmonella* reduced the expression of CXCR4 in a dose-dependent manner in B16F10 and LL2 cells. Indeed, the expressions of phosphorylation AKT, phosphorylation mTOR, and phosphorylation p70S6K were reduced after *Salmonella* treatment in a dose dependent-manner (Fig. 3). We observed the correlation between CXCR4 and the AKT/mTOR/p70S6K signaling pathway. These results suggested that *Salmonella* might inhibit CXCR4 expression through reducing the activity of AKT/mTOR/p70S6K signaling pathway.

### AKT/mTOR/p70S6K signaling pathway was involved in *Salmonella*-mediated CXCR4 expression

**Figure 4:**
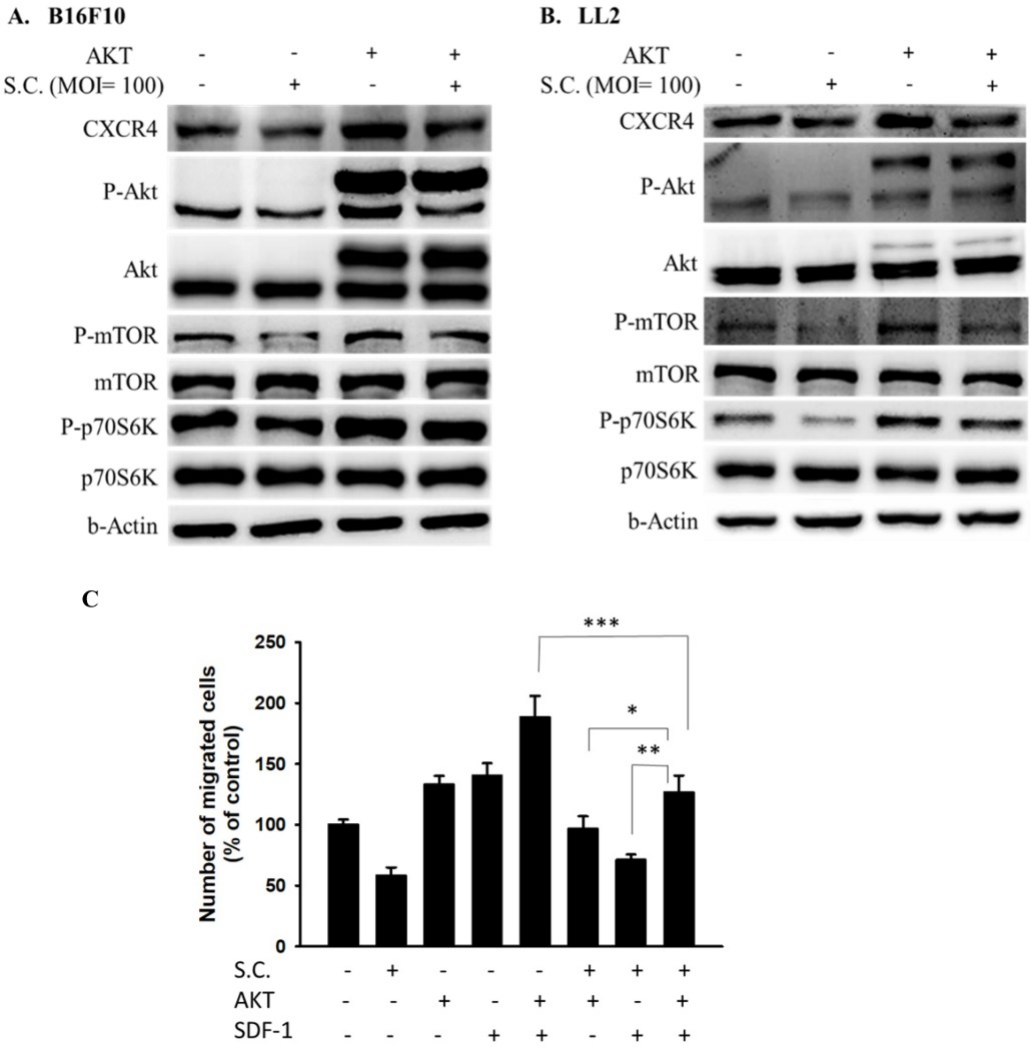
AKT signaling pathways were participated in *Salmonella* (S.C.)-mediated CXCR4 expression. The B16F10 and LL2 cells were transfected with an active AKT plasmid. The cells were treated with* Salmonella* (MOI = 100) for 1.5 h. The various protein expressions in B16F10 and LL2 cells was measured. The immunoblotting assay was repeated three times with similar results. Inserted values indicated relative proteins expression in comparison with β-actin. B16F10 cells infected with *Salmonella* (MOI=100) were added to the upper chamber (with permeable membrane) in the 24-well plates and CXCL12 (100 ng/ml) were added in the lower chamber, then cultured for 24 hours. The B16F10 cells were transfected with an active AKT plasmid. The cells which migrated through the permeable membrane were stained with DAPI and measured by fluorescensce microscope (200X). (n = 6, mean ± SD. *p < 0.05; ** p < 0.01; *** p < 0.001). These replicates were used different passage of cells.

In this study, *Salmonella* reduced the phosphorylation AKT and the expression of CXCR4. Dependent on our previous studies, the constitutively active AKT plasmids can be used to distinguish the correlation between targeting protein and the AKT/mTOR signaling pathway 6. The AKT/mTOR/p70S6K signaling pathway was reversed after transfecting constitutively active AKT plasmids (Fig. 4 A and B). The CXCR4 expression was reversed after being treated with *Salmonella* and constitutively AKT in B16F10 and LL2 cells, indicating that the *Salmonella*-mediated decrease of CXCR4 through AKT/mTOR/p70S6K signaling pathway (Fig. 4A and B). We used Transwell assay to evaluate the role of AKT in SDF-1-induced tumor cell migration. As Fig. 4C shown,* Salmonella* significantly reduced the movement of SDF-1-treated B16F10 after transfecting constitutively active AKT plasmids. The results demonstrate that downregulation of phosphorylation AKT/mTOR is required for *Salmonella*-mediated CXCR4 expression in tumor cells.

### *Salmonella* reduced lung tumor nodules

**Figure 5:**
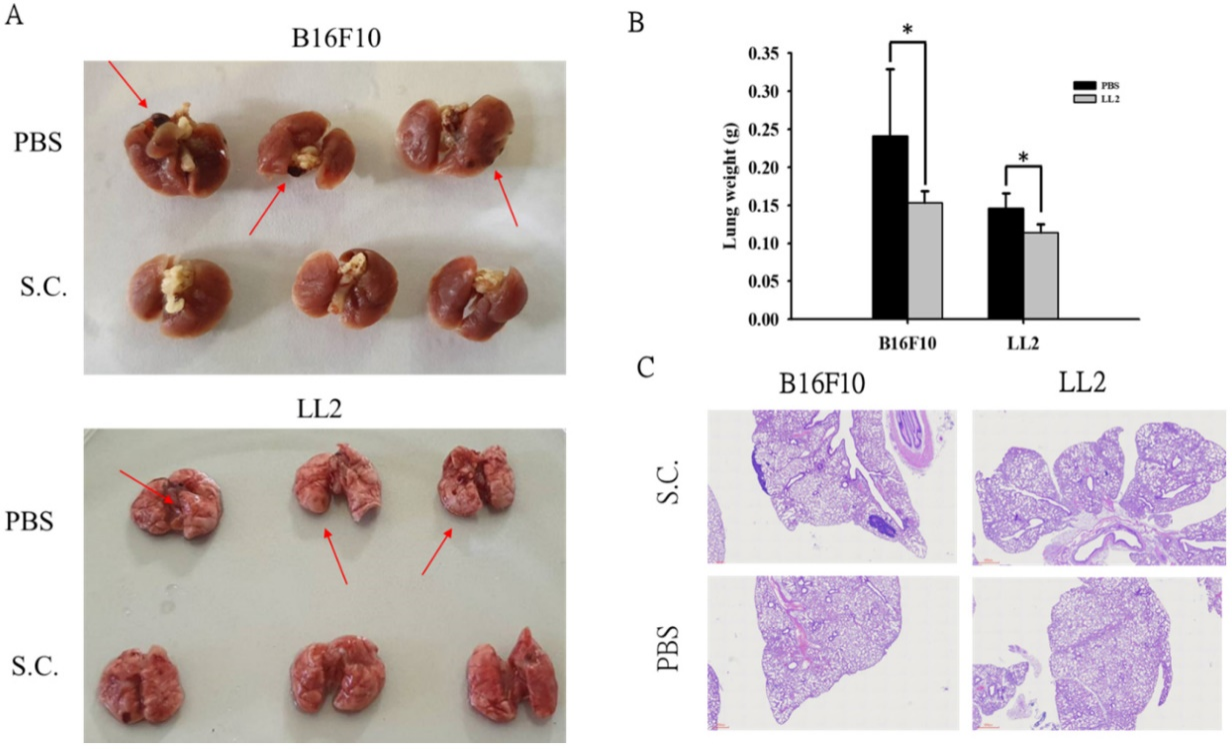
*Salmonella* reduced lung tumor nodules. The tumor cells admixed with *Salmonella* (MOI = 100) for 90 min and then intravenously injected into mice. Mice bearing metastatic nodules were sacrificed after inoculation of the tumors on day 18 day. (**A**) Representative examples of metastatic pulmonary nodules produced 18 days after intravenous injection of tumor cells. (**B**) The anti-tumor effect of *Salmonella* was measured by lung weight. (n = 3, data are expressed as mean ± SD. * p < 0.05) (**C**) Histological examination of pulmonary tumor nodules at day 18 after tumor inoculation (40X).

Moreover, we took advantage of a platform that screens for anti-metastatic molecules 14. The tumor cells admixed with *Salmonella* (MOI = 100) for 90 min and then intravenously injected into mice. Mice bearing metastatic nodules were sacrificed after inoculation of the tumors on day 18 day. The numerous pulmonary nodules were observed in the lungs from PBS-treated mice. However, the smaller and fewer tumor nodules were observed in the lungs from the mice treated with *Salmonella* (Fig. 5A). The weight of wet lung was measured to quantitatively determine tumor burden. The mice injected with B16F10 and LL2 tumor cells admixed with* Salmonella* had 36% and 22% lower wet lung weight, respectively, compared with those injected with cells admixed with PBS (Fig. 5B). Histological examination confirmed the macroscopic findings (Fig. 5C). Taken together, these results suggest that *Salmonella* affects tumor metastasis.

## Discussion

It is surprising that* Salmonella* not only inhibits primary tumor growth but also prevent metastasis. This suggests that *Salmonella*, which inhibits primarily AKT/mTOR signaling pathway, may represent promising a target agent preventing metastasis of many aggressive tumors that use CXCR4 for the guided migration of tumor cells from primary tumors to secondary colonization sites. The SDF-1 is often produced at high levels by metastatic target organs including lung, liver and bone 15. CXCR4 upregulation in tumor cells enhanced the migratory potential of tumor cells towards SDF-1 producing organs 15. The CXCR4-inhibitors a promising class of anti-tumor agents that should be further explored in clinical trials. CXCR4 inhibitors are in clinical use as bone marrow stem cell or progenitor cell mobilizers 16.

Therefore, the ability of *Salmonella* to target multiple tumors from distant sites makes it an ideal anti-tumor agent over some other cancer therapeutic agents limited to local administration. The anti-CXCR4 activity was specific in tumor sites by tumor-targeting *Salmonella*. The special effects of *Salmonella* can inhibit primary tumor growth and reduce metastasis from primary tumor. Previously, *Salmonella* had that activity that is anti-angiogenesis by reducing hypoxia-inducible factor 1-α expression 11. Interestingly,* Salmonella* infected with porcine and analyzed RNA profiles from the mesenteric lymph nodes 17. CXCR4 mRNA was upregulated in this model. *Salmonella* may enhance immune cell response, but reduced the epithelial-mesenchymal transition (EMT) of tumor. *Salmonella* can break tumor immune tolerance and reduce tumor metastasis.

In a tumor microenvironment, two main producers of Interferon-γ (IFN-γ) are natural killer (NK) cells, and tumor-specific cytotoxic CD8+ T lymphocytes (CTLs). Th1 polarized CD4+ T helper cells also secrete IFN-γ that help in the promotion and maintenance of anti-tumor CTL responses. Previously, we showed that *Salmonella* significantly upregulated IFN-γ which may be responsible for recruiting peripheral immune cells to the tumor in wild-type mice, but not in T-cell-deficient mice 18. We suggested the T cell is involved in the regulation of *Salmonella*-induced host antitumor immunity in tumor-bearing mice. Thus, our studies may provide a cellular basis for understanding the recruitment of effector immune cells and the synergism between the oncolytic effect of* Salmonella* and adaptive antitumor immune mechanisms. *Salmonella* in tumor induced signal transduction by multiple mechanisms including competition for nutrients, stimulation of immune response 19. Moreover, toxin from Salmonella may induce the apoptosis of tumor. As Salmonella replication in tumors and subsequent lysis of tumor cells may induce cell-mediated immune responses to tumor cells, higher oncolysis could account, in part, for an increased infiltrate of immune cells in tumors. The cells undergoing bacteria-induced cell death exhibit heterogeneous morphological features.

Taken together, our study suggests that *Salmonella* is sufficient to inhibit primary tumor growth and CXCR4-dependent migration and metastasis *in vivo*.

## Supplementary Material

Supplementary figures.
