# Supplementary material for: Salmonella reduces tumor metastasis by downregulation C-X-C chemokine receptor type 4
Source: Int J Med Sci. 2021 Jun 1;18(13):2835–41. doi: 10.7150/ijms.60439 (PMC8241761; doi:10.7150/ijms.60439)
Supplement: Supplementary file 1 — Supplementary figures. [file ijmsv18p2835s1.pdf]

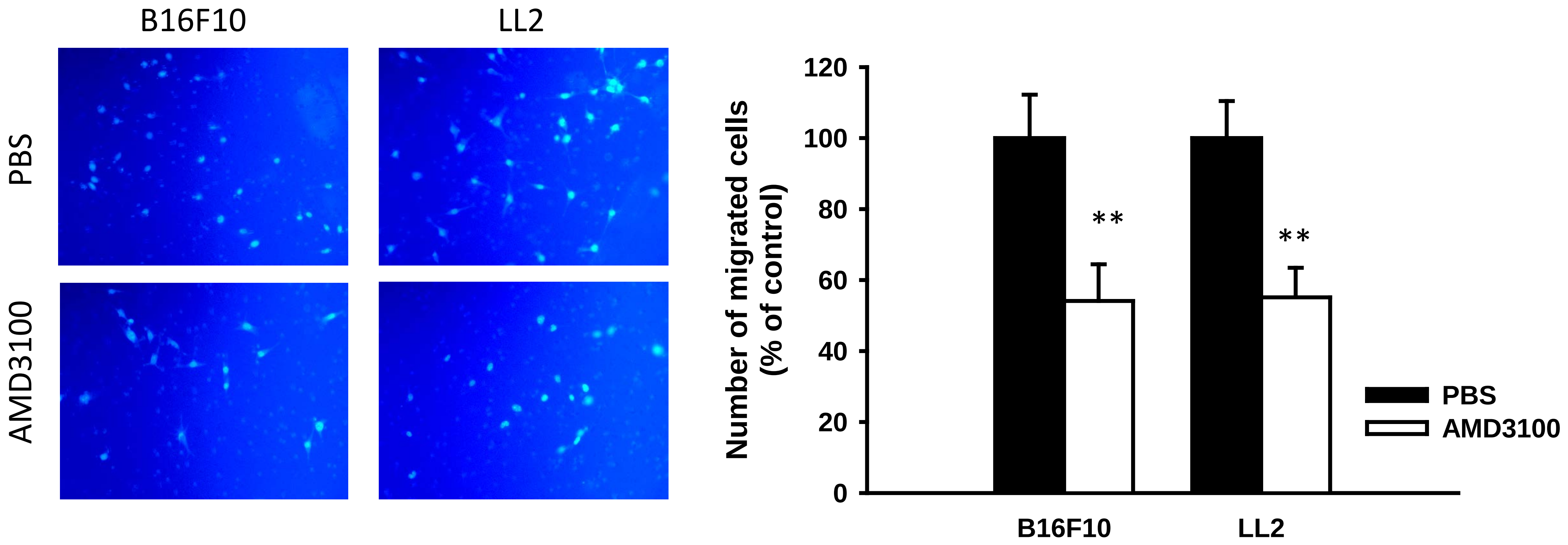

Supplementary Figure 1. The cellular motility of B16F10 and LL2 cells after AMD3100 treatment. The cells were treated with AMD3100 (44nM) for 1.5 h. The B16F10 cells and LL2 cells were placed on the upper layer of Tranwell and then treated with AMD3100 (44nM) for 90 min. After 24 h, the bottom layer of cells were stained with 4',6-diamidino-2-phenylindole (DAPI) and counted under a fluorescence microscope (n = 3, mean ± SD. ** $p < 0.01$). These replicates were used different passage of cells.

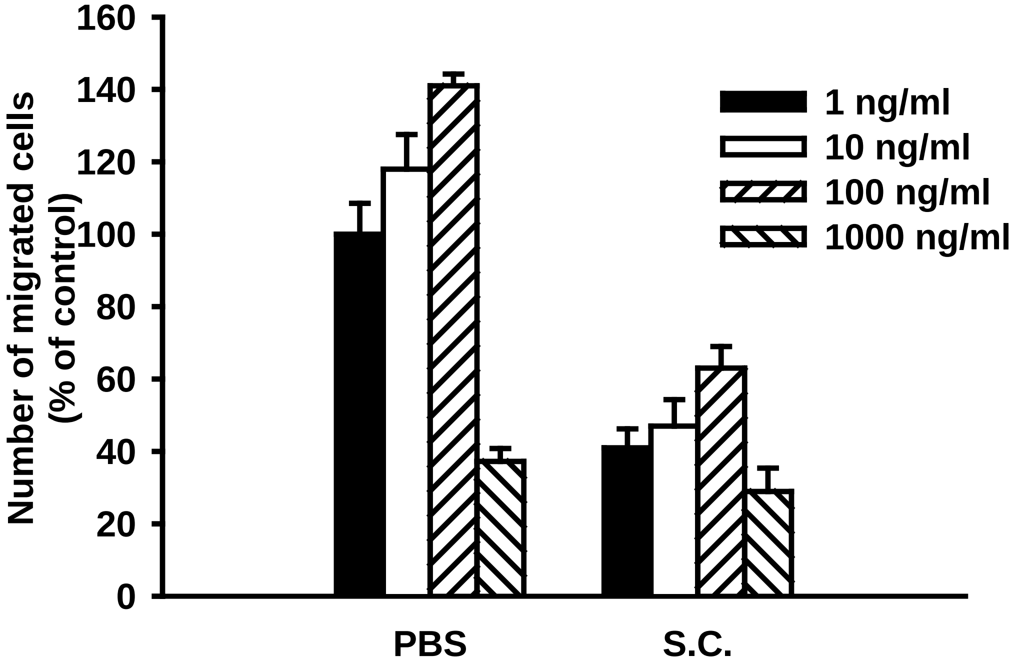

Supplementary Figure 2. Tumor cells infected with Salmonella (MOI=100) were added to the upper chamber (with permeable membrane) in the 24-well plates and SDF-1 (1-1000 ng/ml) were added in the lower chamber, then cultured for 24 hours. The cells which migrated through the permeable membrane were stained with DAPI and measured by fluorescensce microscope (200X).

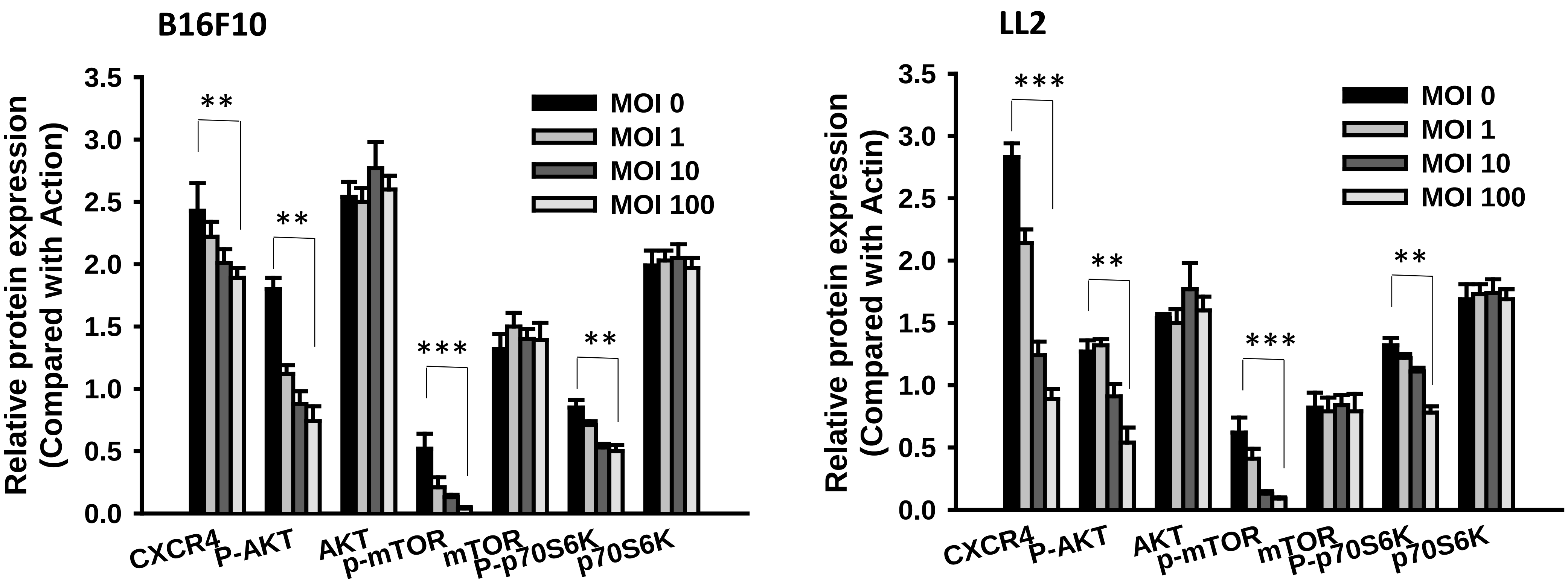

Supplementary Figure 3. Quantified band intensities of respective proteins in Salmonella-infected cell lines. (n = 3, mean ± SD. ** $p < 0.01$ ； *** $p < 0.001$). These replicates were used different passage of cells.

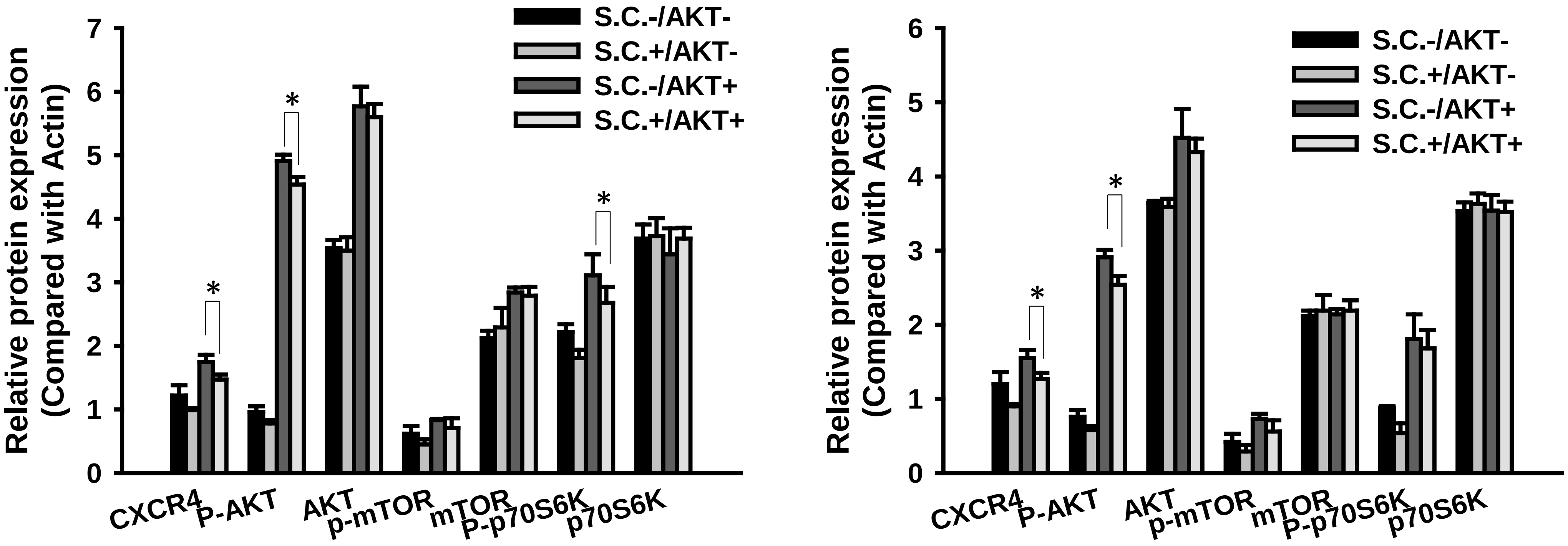

Supplementary Figure 4. Quantified band intensities of respective proteins of tumor cells bearing constitutively active-AKT. (n = 3, mean ± SD. * $p < 0.05$). These replicates were used different passage of cells.
